# Is There Any Difference between the *In Situ* and Systemic IL-10 and IFN-γ Production when Clinical Forms of Cutaneous Sporotrichosis Are Compared?

**DOI:** 10.1371/journal.pone.0162764

**Published:** 2016-09-13

**Authors:** Fernanda N. Morgado, Armando O. Schubach, Maria Inês Pimentel, Marcelo R. Lyra, Érica C. F. Vasconcellos, Claudia M. Valete-Rosalino, Fátima Conceição-Silva

**Affiliations:** 1 Laboratório de Imunoparasitologia, Instituto Oswaldo Cruz/FIOCRUZ, Rio de Janeiro, RJ, Brazil; 2 VigiLeish-Serviço de Infectologia, Instituto Nacional de Infectologia Evandro Chagas/FIOCRUZ, Rio de Janeiro, RJ, Brazil; 3 Departamento de Otorrinolaringologia-Oftalmologia/Faculdade de Medicina/Universidade Federal do Rio de Janeiro, Rio de Janeiro, RJ, Brazil; Mie University Graduate School of Medicine, JAPAN

## Abstract

Fungus of the *Sporothrix schenckii* complex can produce skin lesions in humans, commonly lymphocutaneous (LC) and fixed (F) forms of sporotrichosis. Some authors have suggested that clinical forms are influenced by differences in virulence and genetic profile of isolates. But little is known about the role of immune response in determining the clinical outcome of sporotrichosis. To verify the profile of systemic and in situ IFN-γ and IL-10 expression in sporotrichosis patients, and consequently to detect any difference between the two compartments and/or clinical presentation, we quantified the number of IFN-γ and IL-10 producer peripheral blood mononuclear cells stimulated with *S*. *schenckii* antigen (Ss-Ag) by Elispot, and quantified cytokines expression by *in situ* immunohistochemistry in the same patient. Three groups were formed: 1- LC (n = 9); 2- F (n = 10); 3- healthy individuals (n = 14). All sporotrichosis patients produced high amounts of systemic IFN- γ when compared to uninfected individuals. No differences were observed between LC and F groups. Regarding *in situ* IL-10 expression, a difference between LC and F groups was observed: LC lesions presented higher amounts of IL-10 than F lesions differently from systemic IL-10 which showed similarities. Our data suggests that LC lesions present higher IL-10 expression which could be related to regulatory mechanisms for compensating the tissue injury, however favoring fungal persistence in the lesions. Surprisingly, there were no differences in systemic and *in situ* IFN- γ expression between CL and F patients, although it was significantly higher expressed in these patients than in healthy individuals.

## Introduction

Sporotrichosis is a subcutaneous mycosis caused by fungus of the *Sporothrix schenckii* complex [1–3]. Infection occurs by traumatic inoculation of the fungus in individuals working with soil and plants [4] or through the bite or scratch of sick cats [4–7].

After fungus inoculation, the individual may develop different clinical forms, varying from localized skin lesions (fixed—F) or lymphocutaneous (LC) forms, to systemic disease (extracutaneous form) [4]. About 75% of patients present skin lesions, mainly LC and F forms [4]. The F form is characterized by one lesion (verrucous, ulcerated or plaque-like) on the site of the fungus inoculation without lymphatic involvement. But, the most common form is the LC which is characterized by an involvement of the lymphatic system, accompanied by the occurrence of subcutaneous nodules that may progress to necrosis, liquefaction of their content and ulceration, showing the aspect known as sporotrichoid [4–8]. Although some authors have suggested that clinical forms are influenced by differences in virulence [9] and genetic profile of isolates [10], little is known about immune response in human SP.

Previously, our group [11] had verified differences in the *in situ* immune response between LC and F lesions suggesting that the clinical presentation could be influenced by the different profile of the *in situ* immune response. However, as *Sporothrix* sp can spread all over the body, the role of systemic immune response in immunopathology of sporothrichosis, particularly cytokine profile, cannot be neglected. Based on these facts and aiming at understanding some aspects of the IFN-γ/IL-10 profile in the different clinical presentations and tissue compartment of human sporotrichosis, we quantified the systemic and *in situ* IFN-γ and IL-10 expressions by immunohistochemistry and Elispot assay.

## Materials and Methods

### Ethics statement

An informed written consent was obtained from all individuals. In the case of minor/children, the written consent was obtained from the next of kin, caretakers or guardians. Ethical approval was obtained from the institutional Ethics Committee on Human Research—FIOCRUZ (CEP-INI protocol 014/2001).

### Patients

Nineteen sporotrichosis patients treated at the Instituto Nacional de Infectologia Evandro Chagas—INI/FIOCRUZ and were grouped as follows: 1- lymphocutaneous form (LC; n = 9); and 2- fixed cutaneous form (F; n = 10). Fourteen healthy donors were included in this study. Peripheral venous blood was collected in heparin from all analyzed individuals and lesion fragments were collected from 14 patients. All samples used in this study were obtained at the moment of diagnosis, so the patients were free of medication. After the sample uptake, the diagnosis was confirmed by fungal isolation in appropriate culture media and according to the diagnosis, they were treated by the medical staff of Instituto Nacional de Infectologia Evandro Chagas—Fiocruz.

### Antigen preparation (Ss-Ag)

The yeast-like phase of the *Sporothrix schenckii* (Ss) strain 17629, kindly provided by Dr Zancopé-Oliveira (INI-FIOCRUZ) was cultured in brain heart infusion (BHI) medium as described by Brito et al. [9]. Fungal cells were adjusted to 10^8^ cells/mL and disrupted by cycles of freezing and thawing, followed by ultra-sonication (Lab-line Ultra-Lip Labsonic Systems IL, USA).

### Separation of peripheral blood mononuclear cells (PBMC)

PBMC were obtained as previously described [12]. Briefly, PBMC cells were separated by centrifugation over a Ficoll-Hypaque gradient (Histopaque 1077, Sigma, MO, USA) and adjusted to 2x10^6^ cells / mL and 1x10^6^ cells / mL.

### IFN-γ and IL-10 Elispot

The Elispot assay was performed as previously described [13]. Briefly, 96-well microplates (Multiscreen Millipore, France) were coated with 5μg/mL of antibodies anti-IFN-γ or anti-IL-10 (Mabtech, OH, USA). A total of 2x10^5^ and 1x10^5^ PBMC were added to the ELISPOT plates in the presence of medium alone (spontaneous cytokine production), Ss-Ag (the equivalent of 10^6^ disrupted fungal cells/well) or Concanavalin A (ConA) (4μg/mL–Sigma). After stimulation, the plates were washed and incubated with either 1μg/mL biotinylated anti-IFN-γ or anti-IL-10 (MabTech). The plates were incubated with streptavidin-alkaline phosphatase (MabTech) and revealed with BCIP/NBT (Sigma). The spots were counted in an Immunospot reader [Cellular Technology (CTL) Ltd, OH, USA] using the Immunospot Software Version 3 (CTL).

The results were expressed as spots/10,000 PBMC in the antigen stimulated wells subtracted of the spontaneous expression.

### Immunohistochemistry

The *in situ* inflammation was evaluated by immunoperoxidase staining as described [14]. The primary antibodies used were: rat anti- human IL10 (clone JES3-12G8) and rat anti- human IFN-γ (clone XMG1-2) (BD Biosciences, CA, USA), diluted 1:100 in PBS (phosphate buffered saline). Suppressed primary antibodies was used as negative control. The images were acquired using the Motic Images Plus2.0 program (Motic China Co., China) and light microscopy (Nikon eclipse E200, Japan). The staining intensity was scored in ten microscopic fields (200x magnification) as rare (at least 1 positive area / field), discrete (2–3 positive areas / field), moderate (4–5 positive areas / field) and intense (>5 positive areas / field).

### Statistical analysis

The SPSS16 for windows (SPSS, Inc., IL, USA), was used for statistical analysis. Data were reported as median and range and the nonparametric Mann-Whitney test and Fisher`s exact test were used to compare the LC, F and healthy groups. Spearman test was used for nonparametric correlations. P values ≤ 0.05 were considered as positive.

## Results

### Patients

Clinical data are described in Table 1 and S1 table.

**Table 1:** Clinical data of patients with lymphocutaneous (LC) and fixed (F) forms of sporothrichosis.

Clinical data		Fixed	Lymphocutaneous	P
Gender*	Female	7	4	0.37
	Male	3	5	
Mean Age (years)** **(Range)**		35.5	40	0.71
		(9–79)	(19–59)	
**RJ regions**	Rio de Janeiro	7	3	
	Duque de Caxias	1	1	
	São João de Meriti	0	1	
	Nova Iguaçu	1	0	
	Itaguaí	0	1	
	Nilópolis	1	0	
	Saquarema	0	2	
Mean Disease duration (days)** **(range)**		35	40	0.62
		(15–120)	(20–90)	
Mean Number of lesions** **(range)**		1	2	0.09
		(1–3)	(1–6)	
Mean Duration of treatment (days)** **(range)**		67.5	75	0.83
		(45–150)	(30–120)	

*Fisher`s exact test

**Mann-Whitney test

RJ regions = municipalities of Rio de Janeiro state

A p value ≤ 0.05 was considered to be significant

### Histopathology

Suppurative granulomatous inflammation was the predominant finding (7/9) in fixed lesions; the remaining (2/9) presented only granulomatous inflammation. LC lesions presented suppurative granulomatous inflammation (4/8) and necrotic granulomatous inflammation (4/8). Yeast cells compatible with *Sporothrix schenckii* complex were observed in three LC and in four F cases. No differences were observed between groups (p>0.05). Histopathological analysis was not possible in 2 patients due to insufficient material.

### IFN-γ expression by PBMC

**Fig 1:**
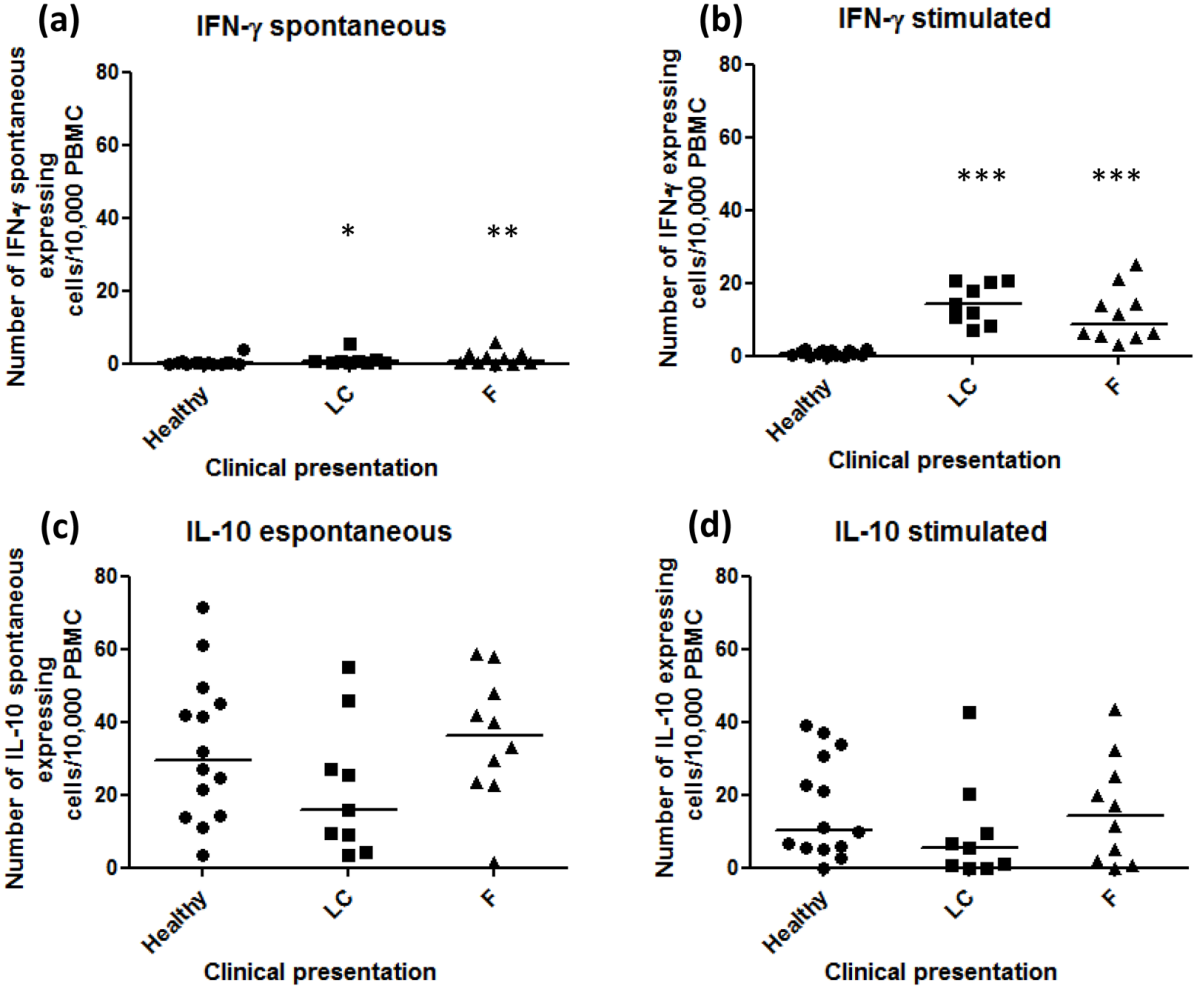
Number of IFN-γ and IL-10 producer cells/2 x 10^5^ cells from peripheral blood. (A) Spontaneous IFN-γ producing cells and (B) IFN-γ producing cells after Ss-Ag stimulation. (C) Spontaneous IL-10 producing cells and (D) IL-10 producing cells after Ss-Ag stimulation. The spontaneous values were subtracted from total spots detected in stimulated cultures. LC—lymphocutaneous sporotrichosis; F- fixed form of sporotrichosis; * p-value = 0.013; **p-value = 0.026; ***p-value = 0.0001 (Mann-Whitney test).

The amount of spontaneous IFN-γ producing cells was lower in the healthy group (median 0.11, 0–3.84) than in LC (median 0.5, 0.1–5.5; p = 0.013) and F patients (median 0.83, 0–5.92; p = 0.026) (Fig 1a and S1 Table). All subjects presented positive response to ConA stimulation (data not shown). Regarding Ss-Ag stimulation, LC (median 14.1, 7–20.3) and F (median 8.8, 3.1–25) patients presented similar quantities of IFN-γ-producer cells (p>0.05), but they were higher than in the healthy group (median 0.65, 0–2, p = 0.0001 and 0.0001, respectively) (Fig 1b). An inverse correlation between spontaneous IFN-γ-producer cells and duration of lesions was observed (r = -0.578; p = 0.008).

### IL-10 expression by PBMC

Healthy subjects showed substantial amounts of spontaneous IL-10 expression (median 29.45, 3.3–71.6) and they were similar to LC (median 15.7, 3.5–55.0) and F patients (median 36.45, 1.6–58.7; p>0.05) (Fig 1c and S1 Table).

Ss-Ag was able to stimulate IL-10 expression by PBMC from the majority of subjects (LC: median 5.4, 0–42.7; F: median 14.35, 0–43.4; Healthy: median 10.45, 0–39) (p>0.05) (Fig 1d).

An inverse correlation between spontaneous IL-10 producer cells and the number of lesions was observed (r = -0.542; p = 0.017).

### IFN-γ *in situ* expression

**Fig 2:**
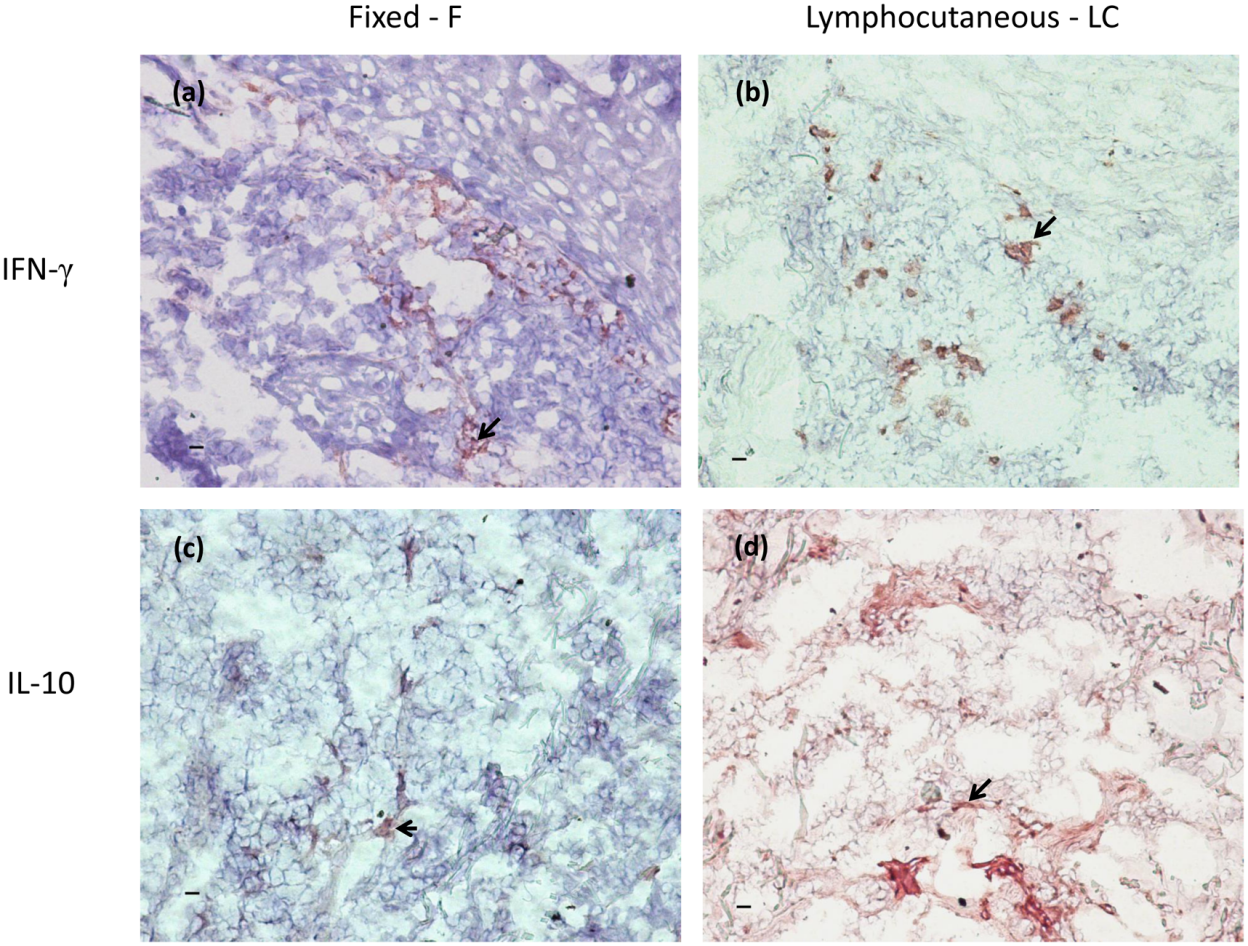
*In situ* IFN-γ and IL-10 expression in lymphocutaneous and fixed lesions of sporothrichosis. The *in situ* IFN-γ and IL-10 expression was detected by immunohistochemistry. The arrows point positive areas (red/AEC– 3-amino-9-ethylcarbazole). The intensity of staining was scored in ten microscopic fields (200x magnification) as rare (at least 1 positive area / field), discrete (2–3 positive areas / field), moderate (4–5 positive areas / field) and intense (>5 positive areas / field). Scale bar = 10μm.

The distribution of the *in situ* IFN-γ in LC was as follows: three patients with rare, one with discrete, one with moderate and one patient with intense expression. The distribution in F was: three patients with rare, one with discrete and four patients with moderate expression. No difference was observed when groups were compared (p = 0.627; OR = 2.00) (Fig 2a and 2b and Table 2 and S1 Table).

**Table 2:** *In situ* IFN-γ expression in lymphocutaneous (LC) and fixed (F) forms of sporotrichosis.

Clinical presentation	Rare to discrete	Moderate to intense	Total
**Lymphocutaneous**	4	2	6
**Fixed**	4	4	8
**Total**	8	6	14

p > 0.05 Fisher’s exact test.

### IL-10 *in situ* expression

The *in situ* IL-10 expression was distributed in LC as follows: one patient with rare, two with moderate and three patients with intense expression. The distribution in F was: two patients with rare, five with discrete and one patient with moderate expression. The LC group presented higher IL-10 expression than the F patients (p = 0.03; OR = 0.028) (Fig 2c and 2d and Table 3 and S1 Table). In addition, an association between higher IL-10 *in situ* expression by cells in LC lesions and necrotic granulomatous inflammation was observed.

**Table 3:** *In situ* IL-10 expression in lymphocutaneous (LC) and fixed (F) forms of sporotrichosis.

Clinical presentation	Rare to discrete	Moderate to intense	Total
**Lynphocutaneous**	1	5	6
**Fixed**	7	1	8
**Total**	8	6	14

p = 0.03; OR = 0.028 (Fisher’s exact test).

## Discussion

In the present study, to detect differences in IFN-γ/IL-10 profile in different clinical presentations of human sporotrichosis, we quantified spontaneous and antigen stimulated IFN-γ and IL-10 producer cells from peripheral blood and in skin lesions of patients with lymphocutaneous (LC) and fixed (F) forms of sporotrichosis. Although relevantly expressed, IFN-γ and IL-10 systemic expression cells (peripheral blood) was similar when the two clinical forms were compared. On the other hand, we could detect a higher IL-10 *in situ* expression by cells in LC lesions, associated with necrotic granulomatous inflammation and a tendency of higher number of lesions, longer duration and longer treatment duration. Our previous data showed that, when compared with F group, LC lesions presented stronger inflammatory profile with higher number of lesions, longer duration and required longer treatment duration, as well as more *in situ* CD4^+^ cells, neutrophils, and more intense NOS2 expression [11]. Macrophage concentration was similar and comprised about one third of all cells in sporothrichosis lesions. In this context, the high IL-10 expression now verified could play a role in regulatory mechanisms to control/modulate the tissue injury observed in these lesions. IL-10 can be produced by alternatively activated macrophages (M2) [15]. These diverse physiological functions result from the remarkable plasticity of macrophages, which allows these cells to dramatically change their form and function in response to local environmental signals or tissue damage [16]. Furthermore, the subtype M2b (IL-10 and TNF-α producer) was correlated to increased susceptibility to gastrointestinal candidiasis in murine model [17]. Recently, the *in vitro* induction of M2 by *Sporothrix schenckii’*s cell wall molecules was demonstrated [18]. Mice experimentally infected presented high IL-10 which was associated with the apoptotic process induced by the fungal infection [19].

The detection of systemic and *in situ* IFN-γ production cells in LC and F patients suggested a role of IFN-γ in the inflammation of cutaneous sporotrichosis. The induction of Th1 response along with abundant IFN-γ production in an *in vitro* study [20] in mice was achieved by fungal isolates from patients presenting cutaneous manifestations. In addition, the resistance of NOS2 knockout mice was related to a higher IFN-γ production and a reduced spontaneous and antigen stimulated IL-10 expression in wild type mice [21]. Altogether, these data suggested that the role of IFN-γ in fungal burden control and development of benign clinical presentations could not be related to macrophage activation via NOS2. Furthermore, the authors also suggested that although NO was an essential mediator to the *in vitro* killing of *S*. *schenckii* by macrophages, NO *in vivo* could contribute to the immunosuppression and cytokine balance during the early phases of infection [21].

In experimental sporotrichosis and *in vitro* studies using isolates from visceral and cutaneous origins it was established that Th1 response mostly IFN-ƴ, which is produced by different cells including CD4 T cells, strongly activates macrophages and thereby innate and adaptive immune responses and perhaps determines its clinical manifestations[20,22–24]. Herein, we observed a markedly IFN-ƴ expression in active lesions that did not differ between two different cutaneous manifestations, probably suggesting that Th1 cells were predominant at the lesions site. However, other factors could be taking place in LC lesions favoring fungal persistence and dissemination. In this context, intrinsic regulations, as the elevated IL-10 expression observed herein could favor the development of extensive lesions in lymphocutaneous patients. In addition, the possibility of extrinsic regulations such as the use of anti-TNF-alpha monotherapy favoring the lesion severity was described[25].

Zhang et al [26] showed large IL-10 expression induced by components of *S*. *schenckii* in rats experimentally infected. An induction of a Th2 profile with IL-10 and IL-4 production by *S*. *schenckii* isolated from visceral origins was observed, suggesting that visceral isolates may escape from the host immune response by inducing little Th1-prone responses [20]. The role of Th2 cells was also described by Maia et al (2006)[24]. Maia et al 2006 studied a murine model of disseminated sporotrichosis and observed alternation between Th1 and Th2 responses according to infection evolution. Curiously, they observed that Th2 response coincided with high NO production and NOS2 expression and elevation in fungal burden[24]. Some authors have demonstrated the influence of wall compounds of fungal cells on immune response [27–29]. The lipid wall compound was found to inhibit the phagocytosis and to induce high liberation of NO and TNF-α in macrophage cultures [28]. Furthermore, the lipid extract could be recognized by TLR4 which induces IL-10 and TNF expression by peritoneal macrophages, as well as NO production, all associated with clinical worsening [30]. This suggests that *S*. *schenckii* could be able to modulate the immune response also by inducing IL-10 production by human cells, probably as a mechanism of immune escape. Maia et al. [19] demonstrated that the ExoAntigen was able to stimulate IL-10 production in *in vitro* infected mice. We observed that Ss-Ag stimulated IL-10 expression by PBMC from both healthy and patient groups. Thus, IL-10 could influence fungal clearance allowing the antigen persistence and consequently favoring the worsening of the lesion. On the other hand, we cannot exclude the possibility of a cross reaction between Ss-Ag and antigens from other fungal agents, particularly those that compose the normal microbiota like those with similar PAMPs [31,32]. The influence of *Candida* sp cell wall in the expression of inflammatory and regulatory cytokines has been described [33,34]. β-glucans are frequently observed in cell walls of several fungi [35] including *Candida* sp and *S*. *schenckii* [36] and can induce IL-10 expression causing subversion of immune response [35]. Moreover, the possibility of lipopolysaccharide (LPS) contamination of Ss-Ag can be rejected since PBMC from healthy subjects produced insignificant amounts of IFN-spots under Ss-Ag stimulation.

Spontaneous IFN-γ-producing PBMC from patients were higher than from healthy subjects. The present data supports previous results which indicate that despite the inflammatory activity being concentrated at the lesion site, the immune response can be reflected at the peripheral system as observed in the peripheral blood compartment, and it can be specifically measured. Systemic reactions like erythema nodosum have already been verified in sporothrichosis patients presenting only cutaneous lesions, along with fever, arthralgia and malaise [37]. As fungal cells were not verified in tissues from erythema nodosum, Gutierrez-Galhardo et al. [37] suggested that these systemic reactions could play a protective role, leading to a more benign evolution of the disease.

Although the small number of cases could be considered as a limitation of this study, this is the first publication comparing the *in situ* and systemic cytokine expressions in human sporotrichosis. Moreover, studies with similar numbers of human cases have been published in the literature[11,14,38–41].

In conclusion, our data suggests that: 1- LC lesions present higher IL-10 expression which can be related to regulatory mechanisms for compensating the tissue injury, however favoring fungal persistence in the lesions. 2- Surprisingly, there were no differences in systemic and *in situ* IFN- γ expression between CL and F patients, although it was significantly higher expressed in these patients than in healthy individuals. 3-*S*. *schenckii* is able to modulate the immune response also by inducing IL-10 production by human cells probably using this as a mechanism of immune escape.

## Supporting Information

S1 Table Raw data of patients and healthy donors evaluated. (DOCX)
