# Supplementary material for: Is There Any Difference between the In Situ and Systemic IL-10 and IFN-γ Production when Clinical Forms of Cutaneous Sporotrichosis Are Compared?
Source: PLoS One. 2016 Sep 13;11(9):e0162764. doi: 10.1371/journal.pone.0162764 (PMC5021344; doi:10.1371/journal.pone.0162764)
Supplement: S1 Table — (DOCX) [file pone.0162764.s001.docx]

**Support table 1 – Raw data of patients and healthy donors evaluated.**

| **Patient** | **Clinical form** | **Gender** | **Age (years)** | **Disease duration (days)** | **Number of lesions** | **Days of treatment** | **Systemic spontaneous IFN-ƴ *** | **Systemic IFN-ƴ after stimulation*** | ***In situ* IFN-ƴ**** | **Systemic spontaneous IL-10*** | **Systemic IL-10 after stimulation*** | ***In situ* IL-10**** |
| --- | --- | --- | --- | --- | --- | --- | --- | --- | --- | --- | --- | --- |
| **1** | LC | F | 40.0 | Missing | 2.0 | 120.0 | 0.4 | 10.7 | ND | 25.3 | 0.95 | ND |
| **2** | LC | F | 45.0 | 40.0 | 4.0 | 60.0 | 1.10 | 20.3 | ND | 8.9 | 0.6 | ND |
| **3** | LC | F | 39.0 | 60.0 | 6.0 | 106.0 | 0.10 | 7.0 | ND | 3.5 | 42.7 | ND |
| **4** | LC | F | 24.0 | 90.0 | 1.0 | 120.0 | 0.50 | 18.0 | 4.0 | 45.8 | 5.4 | 3.0 |
| **5** | LC | M | 59.0 | Missing | 1.0 | 90.0 | 0.80 | 20.7 | 1.0 | 27.0 | 0.0 | 1.0 |
| **6** | LC | M | 19.0 | 60.0 | 2.0 | 40.0 | 0.10 | 14.1 | 1.0 | 4.08 | 6.6 | 4.0 |
| **7** | LC | M | 46.0 | 20.0 | 3.0 | 30.0 | 0.40 | 11.9 | 2.0 | 15.7 | 9.3 | 4.0 |
| **8** | LC | M | 45.0 | 40.0 | 1.0 | Missing | 0.60 | 20.5 | 1.0 | 9.3 | 20.4 | 3.0 |
| **9** | LC | M | 29.0 | Missing | 1.0 | 30.0 | 5.50 | 8.2 | 3.0 | 55.0 | 0.0 | 4.0 |
| **10** | Fixed | F | 9.0 | 20.0 | 1.0 | Missing | 5.92 | 5.3 | 3.0 | 32.9 | 11.5 | 3.0 |
| **11** | Fixed | F | 11.0 | 120.0 | 1.0 | 60.0 | 0.0 | 6.2 | ND | 1.6 | 32.3 | ND |
| **12** | Fixed | F | 55.0 | 90.0 | 1.0 | 45.0 | 0.2 | 6.2 | 2.0 | 58.7 | 25.0 | 2.0 |
| **13** | Fixed | F | 31.0 | 30.0 | 1.0 | 60.0 | 0.3 | 13.9 | 3.0 | 42.0 | 0.0 | 2.0 |
| **14** | Fixed | F | 46.0 | 40.0 | 3.0 | 100.0 | 2.75 | 3.1 | 3.0 | 23.6 | 1.8 | 2.0 |
| **15** | Fixed | F | 40.0 | 21.0 | 2.0 | 45.0 | 1.35 | 21.0 | 3.0 | 40.0 | 0.8 | 2.0 |
| **16** | Fixed | F | 79.0 | 120.0 | 1.0 | 150.0 | 0.05 | 14.3 | 1.0 | 29.6 | 4.9 | 1.0 |
| **17** | Fixed | M | 16.0 | 30.0 | 1.0 | 75.0 | 2.5 | 5.0 | 1.0 | 58.0 | 43.4 | 2.0 |
| **18** | Fixed | M | 50.0 | 60.0 | 1.0 | 80.0 | 0.3 | 25.0 | ND | 48.0 | 19.7 | ND |
| **19** | Fixed | M | 19.0 | 15.0 | 1.0 | Missing | 1.68 | 11.4 | 1.0 | 22.7 | 17.2 | 1.0 |
| **20** | Healthy | F | 39.0 | N/A | N/A | N/A | 0.24 | 1.2 | N/A | 41.4 | 4.9 | N/A |
| **21** | Healthy | F | 23.0 | N/A | N/A | N/A | 0.56 | 1.44 | N/A | 41.8 | 0.0 | N/A |
| **22** | Healthy | F | 24.0 | N/A | N/A | N/A | 0.12 | 0.8 | N/A | 45.0 | 6.8 | N/A |
| **23** | Healthy | F | 29.0 | N/A | N/A | N/A | 3.84 | 0.0 | N/A | 71.6 | 5.4 | N/A |
| **24** | Healthy | F | 27.0 | N/A | N/A | N/A | 0.0 | 0.1 | N/A | 26.9 | 39.0 | N/A |
| **25** | Healthy | F | 56.0 | N/A | N/A | N/A | 0.12 | 1.6 | N/A | 32.0 | 9.7 | N/A |
| **26** | Healthy | F | 33.0 | N/A | N/A | N/A | 0.0 | 0.0 | N/A | 11.2 | 21.2 | N/A |
| **27** | Healthy | F | 26.0 | N/A | N/A | N/A | 0.0 | 0.2 | N/A | 3.3 | 5.9 | N/A |
| **28** | Healthy | F | 21.0 | N/A | N/A | N/A | 0.0 | 0.1 | N/A | 24.6 | 37.2 | N/A |
| **29** | Healthy | F | 25.0 | N/A | N/A | N/A | 0.1 | 0.0 | N/A | 21.4 | 11.2 | N/A |
| **30** | Healthy | M | 22.0 | N/A | N/A | N/A | 0.2 | 1.92 | N/A | 49.6 | 2.6 | N/A |
| **31** | Healthy | M | 21.0 | N/A | N/A | N/A | 0.0 | 1.3 | N/A | 14.1 | 22.8 | N/A |
| **32** | Healthy | M | 26.0 | N/A | N/A | N/A | 0.0 | 0.5 | N/A | 61.1 | 33.7 | N/A |
| **33** | Healthy | M | 30.0 | N/A | N/A | N/A | 0.3 | 2.0 | N/A | 14.0 | 30.7 | N/A |

**^N/A- not applicable^**

**^LC – lymphocutaneous form^**

**^M- male^**

**^F – female^**

**^*number of spots/10,000 PBMC^**

**^**Legend: 1- rare; 2- discrete; 3- moderate; 4- intense^**

**^ND - not done^**

**^Missing - data not available^**
